# Detection of SARS-CoV-2 infection clusters: The useful combination of spatiotemporal clustering and genomic analyses

**DOI:** 10.3389/fpubh.2022.1016169

**Published:** 2022-12-01

**Authors:** Yangji Choi, Anaïs Ladoy, David De Ridder, Damien Jacot, Séverine Vuilleumier, Claire Bertelli, Idris Guessous, Trestan Pillonel, Stéphane Joost, Gilbert Greub

**Affiliations:** ^1^Institute of Microbiology, Lausanne University Hospital and University of Lausanne, Lausanne, Switzerland; ^2^Laboratory of Geographic Information Systems (LASIG), School of Architecture, Civil and Environmental Engineering (ENAC), École Polytechnique Fédérale de Lausanne (EPFL), Lausanne, Switzerland; ^3^Group of Geographic Information Research and Analysis in Population Health (GIRAPH), Geneva, Switzerland; ^4^Faculty of Medicine, University of Geneva (UNIGE), Geneva, Switzerland; ^5^Division and Department of Primary Care Medicine, Geneva University Hospitals, Geneva, Switzerland; ^6^La Source School of Nursing, University of Applied Sciences and Arts Western Switzerland (HES-SO), Lausanne, Switzerland

**Keywords:** SARS-CoV-2, COVID-19, epidemiology, spatiotemporal cluster, genomics, public health surveillance, superspreading, genetic similarities

## Abstract

**Background:**

The need for effective public health surveillance systems to track virus spread for targeted interventions was highlighted during the COVID-19 pandemic. It spurred an interest in the use of spatiotemporal clustering and genomic analyses to identify high-risk areas and track the spread of the SARS-CoV-2 virus. However, these two approaches are rarely combined in surveillance systems to complement each one's limitations; spatiotemporal clustering approaches usually consider only one source of virus transmission (i.e., the residential setting) to detect case clusters, while genomic studies require significant resources and processing time that can delay decision-making. Here, we clarify the differences and possible synergies of these two approaches in the context of infectious disease surveillance systems by investigating to what extent geographically-defined clusters are confirmed as transmission clusters based on genome sequences, and how genomic-based analyses can improve the epidemiological investigations associated with spatiotemporal cluster detection.

**Methods:**

For this purpose, we sequenced the SARS-CoV-2 genomes of 172 cases that were part of a collection of spatiotemporal clusters found in a Swiss state (Vaud) during the first epidemic wave. We subsequently examined intra-cluster genetic similarities and spatiotemporal distributions across virus genotypes.

**Results:**

Our results suggest that the congruence between the two approaches might depend on geographic features of the area (rural/urban) and epidemic context (e.g., lockdown). We also identified two potential superspreading events that started from cases in the main urban area of the state, leading to smaller spreading events in neighboring regions, as well as a large spreading in a geographically-isolated area. These superspreading events were characterized by specific mutations assumed to originate from Mulhouse and Milan, respectively. Our analyses propose synergistic benefits of using two complementary approaches in public health surveillance, saving resources and improving surveillance efficiency.

## Introduction

The extreme rapidity of the COVID-19 pandemic revealed the importance of developing, and strengthening, public health surveillance systems at both international, national, and regional levels (1). Defined as “the ongoing, systematic collection, analysis, and interpretation of health data essential to the planning, implementation and evaluation of public health practice” (2), an effective public health surveillance system must be able to monitor the spatial and temporal spread of a disease in a timely manner, to quickly detect emerging clusters of infection and cut chains of transmission (3).

In this context, spatiotemporal approaches that investigate disease clustering, such as prospective space-time scan statistics (4), can constitute an integral part of such surveillance systems by systematically detecting emerging clusters of disease that require further investigations. Fundamentally, space-time scan statistics test whether the number of temporally close cases observed in a defined area exceeds the expected number according to the underlying at-risk population. In the context of the COVID-19 pandemic, several studies investigated how prospective space-time scan statistics could contribute to the ongoing surveillance of the pandemic at different spatial levels including a country-wide investigation using publicly available data across the United States of America (5), as well as investigations at higher spatio-temporal resolutions using laboratory test results to detect COVID-19 clusters in a Swiss state (6) and in New York City (7). A drawback of using these approaches is that they rely on health data that are usually geocoded to a patient's residential location, which constitutes only one part virus transmission. Therefore, it may limit the ability of these scan statistics to depict epidemic trajectories and break the infection transmission chain. Some studies have investigated the interplay between geographical and transmission clusters in the context of sexually transmitted diseases (8, 9), but this research question has not been studied, to our knowledge, in the context of COVID-19.

At the same time, the role of genomics has become critical in the public health domain during the SARS-CoV-2 pandemic. The first SARS-CoV-2 genome sequences allowed the scientific community to characterize the virus and understand its zoonotic origin, infection and transmission mechanisms, as well as COVID-19 pathogenesis (10, 11). Sequencing data also enabled biotechnology companies and pharmaceutical companies to quickly develop molecular diagnostic assays and vaccines. Virus genomes from infected individuals were constantly sequenced and submitted to public national (12) and international (13) databases (e.g., GISAID database), forming hubs for SARS-CoV-2 genomic data sharing that assisted worldwide collaborations and standardized lineages definition (14). In parallel, many open-source bioinformatic tools were actively developed, to compare virus genomes, define and assign lineages, facilitating epidemiological investigations. Based on the plentiful open data and bioinformatic tools, numerous SARS-CoV-2 genome-based studies identified new variants of concern (15–17) and tracked geographic transmission of the virus (18–23) in different countries. Although we found numerous studies tracing the origin and evolution dynamics of the COVID-19 pandemic, very few studies examined how genomic sequencing could be used for informed-decision making within an actionable time frame (24, 25).

In this context, our study aimed to investigate: (i) to what extent clusters identified by space-time scan analysis are confirmed as transmission clusters based on SARS-CoV-2 genome sequences, (ii) how genomic-based approaches can improve the epidemiological investigation associated with spatiotemporal clusters, and (iii) how can a combination of both complementary approaches be used in the context of infectious disease surveillance systems. To answer these questions, we sequenced the SARS-CoV-2 genomes of 172 cases contained in a set of spatiotemporal clusters identified in the Swiss state of Vaud during the first epidemic wave in Switzerland (6). We then analyzed genetic similarity among cases within spatiotemporal clusters and spatiotemporal distribution across virus genotypes using different bioinformatic tools to better understand discrepancies and possible synergies between genomic-based and spatiotemporal clustering approaches.

## Methods

### Study design

We previously described the spatiotemporal spread of COVID-19 during the first wave of the pandemic for the state of Vaud, Switzerland, using a prospective space-time scan analysis (6). Briefly, the analysis was performed on 3,317 individuals who were tested (RT-PCR) positive for SARS-CoV-2 between March 2 and June 30, 2020, geocoded to their residential address. The study was approved by the *Commission cantonale d'éthique de la recherche sur l'être humain (CER-VD)*, Switzerland (n°2020-01302). Spatiotemporal clusters were detected daily by comparing the number of observed cases to the expected number within and outside a circular window of varying sizes. Expected cases were estimated with a Poisson model adjusting for population size at the inhabited hectare level, and the analytical window was defined to contain a maximum of 0.5% of the population at-risk and last a maximum of 14 days.

**Figure 1:**
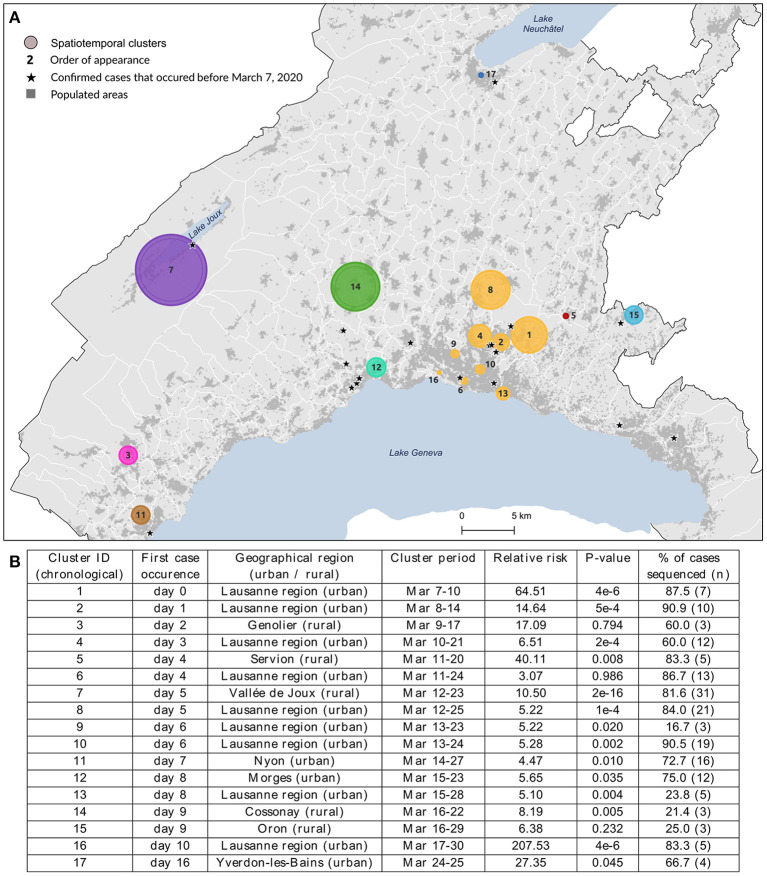
Spatial distribution **(A)** and characteristics **(B)** of the 17 spatiotemporal clusters considered for genomic data analysis. These clusters were identified using a space-time scan statistic run daily from March 2 to June 30 and implemented with SaTScan version 9.6.1 (43). Characteristics include each cluster identifier with its corresponding geographical region, cluster period, relative risk of becoming infected to COVID-19 within the cluster compared to outside, significance evaluated with 999 Monte-Carlo permutations, and the proportion of sequenced cases within cluster. Clusters are colored according to the geographical region to which they belong.

Of the 1,784 spatiotemporal clusters identified (454 with a *p*-value < 0.05), we selected 17 clusters for further investigation (Figure 1). This small number of clusters is partly explained by the many overlapping clusters due to analysis frequency. The selected clusters were chosen to be representatives of the spatial footprint and temporal variations obtained during the first wave of the pandemic, to allow for the comparison of different settings. We chose clusters from different geographical settings (urban vs. rural), of different sizes in terms of geographical coverage and number of cases, as well as some with unique particularities. Additionally, for clusters that were detected several days in a row (i.e., overlapping clusters), we selected the last appearance in order to increase the time span of analysis, even if the last occurrence was not necessarily significant (clusters #3, #6, #15 in Figure 1). The cluster selection process is depicted in Supplementary Figure 1.

### SARS-CoV-2 genome sequencing

We sequenced the SARS-CoV-2 genome of all cases presenting over 10,000 cp/ml from the 17 clusters to investigate the genetic similarity within spatiotemporal clusters. SARS-CoV-2 RNA was extracted from nasopharyngeal swabs (COPAN UTM medium, 3.5 ml) using the MagNA Pure 96 system (Roche, Basel, Switzerland). The viral genomes were amplified by the CleanPlex SARS-CoV-2 panel (Paragon Genomics, SKU 918011) following the manufacturer's instructions (26). The quality of amplified products was assessed by Fragment Analyzer standard-sensitivity NGS (DNF-473; AATI) and quantified using Qubit standard-sensitivity double-stranded DNA (dsDNA) kit (Q32853; Invitrogen). The amplicons were sequenced by 150 bp paired-end reads on a MiSeq (Illumina, San Diego, CA). To evaluate sequencing quality, negative and positive internal controls were included in each run.

### Reads processing and quality control

Reads were processed with GENCOV pipeline (https://github.com/metagenlab/GENCOV), modified from CoVpipe (https://gitlab.com/RKIBioinformaticsPipelines/ncov_minipipe), in order to perform sequence filtering with fastp (27), primer trimming with fgbio (28), mapping to the reference genome NC_045512.2 with bwa (29), alignment evaluation with Qualimap (30), and variant calling with Freebayes (relative number of variant supporting reads = 0.1, minimal depth = 10, absolute number of variant supporting reads = 9) (31). Variants were further filtered by bcftools (32), determining the consensus based on the variants supported by more than 70% of mapped reads, whereas positions covered by fewer than 10 reads were masked with Ns. The consensus sequence was assigned to SARS-CoV-2 lineages using Pangolin (33). The quality of SARS-CoV-2 genome sequences was then manually evaluated according to quality criteria as described by Jacot et al. (34), including mutations supported by 10–70% of mapped reads termed “low-frequency variants”. Genome sequences that did not pass quality criteria were repeated.

### Genomic analyses

Pairwise single nucleotide variant (SNV) distances were computed from quality-checked sequences using Nextstrain SARS-CoV-2 multiple sequence alignment (https://github.com/nextstrain/ncov) (35) and pairsnp (https://github.com/gtonkinhill/pairsnp). Based on the pairwise SNV matrix, we computed the Jaccard similarity index (36) to quantify genetic similarity within spatiotemporal clusters, by calculating the size of the intersection divided by the size of the union of SNVs. Jaccard similarity index was computed for each pair of genomes within the same cluster. Sets of samples with identical SARS-CoV-2 genome sequence (0 SNV distance) were defined as “genomic groups”.

### Genomic and geographic visualization

Phylogenetic analysis and visualization were conducted with Augur and Auspice, respectively, which are parts of Nextstrain that allows for customization and interactive web visualization (35). The relationships among genomic groups and samples with unique genome sequences were visualized as minimum spanning trees (MST) on Cytoscape (37), as demonstrated in Supplementary Figure 2. The network was computed with the optrees package in R (https://github.com/cran/optrees) adopting Prim's algorithm, which finds the shortest path by selecting a subset of the edge such that a spanning tree is formed with the minimal total weight of the edges (38). Each node represents either a genomic group or an individual sequence and the weight of the undirected edges reflects SNVs. The mapping of genomic groups within clusters was done using QGIS 3.22 (QGIS.org, 2022. QGIS Geographic Information System. QGIS Association. http://www.qgis.org).

## Results

### Description of selected spatiotemporal clusters

We investigated the genetic similarity within a set of 17 spatiotemporal clusters selected from a previous study (6). Clusters were detected from March 7 to March 30, 2020, and lasted from 2 to 14 days, corresponding to the lower and upper bound values of the temporal window used in the analysis. The clusters' geographic location and characteristics are shown in Figure 1, where clusters are labeled according to their chronological occurrence. Forty percent of clusters (*n* = 7) were in rural areas or intermediate-size cities, but the first cluster detected (#1) occurred in the Lausanne region, the capital of Vaud state. Cases of COVID-19 had already been declared in Vaud state a few days before the commencement of the study (the first case occurred on March 3), but this did not form any cluster. Their locations are starred in Figure 1A.

While the clusters included 264 lab-confirmed RT-PCR positive cases, only those with a viral load above 10,000 copies/ml (*N* = 172, 65.4%) could be sequenced (see the proportion by cluster in Figure 1B), though this did not affect characterization of the affected populations. The number of cases within clusters varied from 3 to 38 (cluster #7), where individuals were 52.3% female, with a mean age of 57.2 years (σ = 20.2). Detailed characteristics per cluster are provided in Supplementary Table 1. Infected individuals in rural areas tended to be older (median age 73 vs. 54 years, *p*-value < 0.001, Wilcoxon) with a lower mean viral load (230 vs. 590 million copies/ml, *p*-value = 0.04, Wilcoxon) when compared to individuals in urban areas.

The nine clusters within Lausanne metropolitan area (#1, #2, #4, #6, #8, #9, #10, #13, #16, a total of 94 cases) were labeled uniformly as the “Lausanne region” to reduce the complexity of representation. This choice was reinforced by the distinct patterns observed between these urban clusters and the rest of the state.

### Genetic similarity within spatiotemporal clusters

**Figure 2:**
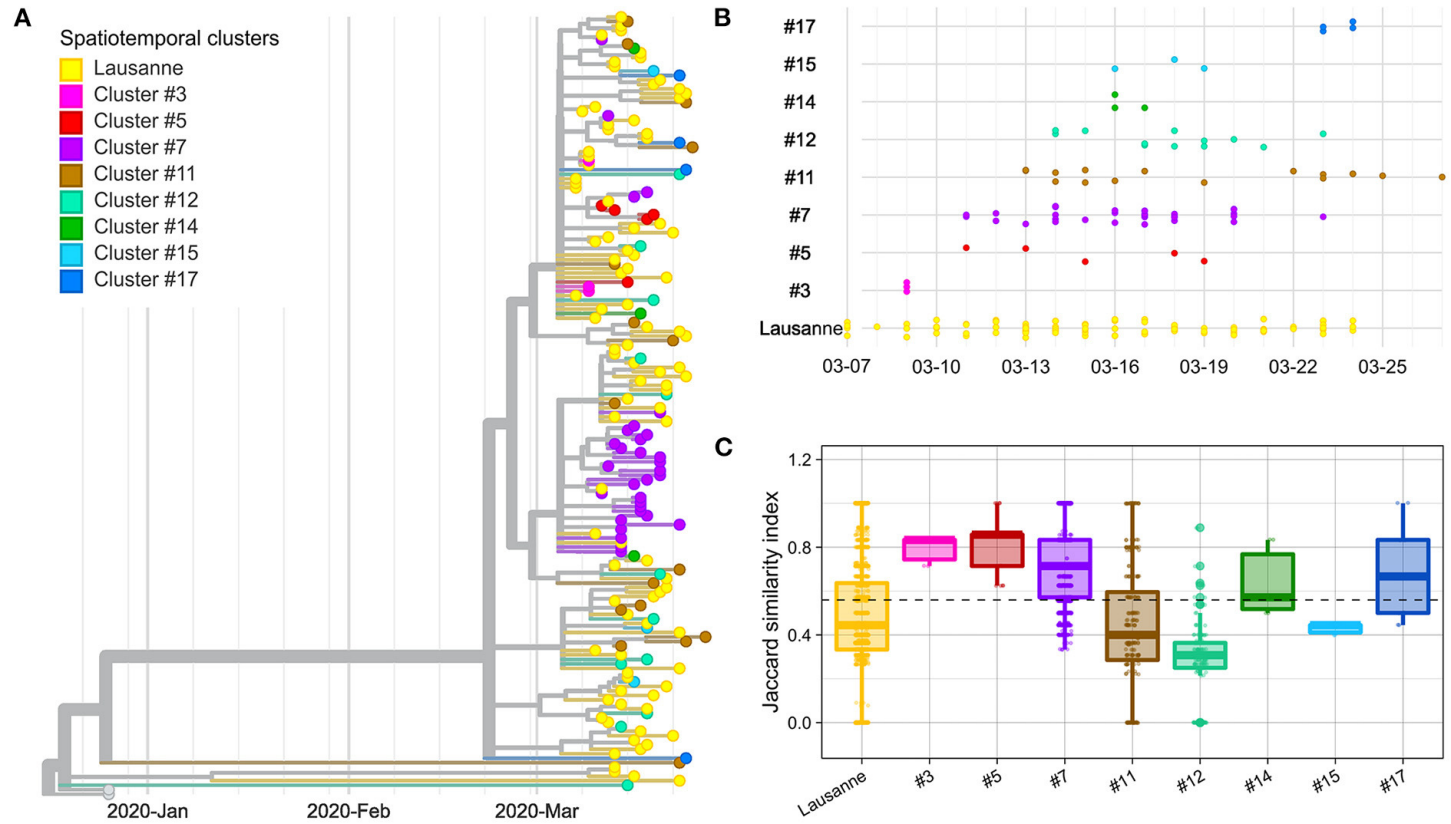
Genetic distance between and within spatiotemporal clusters. **(A)** Phylogenetic tree with 172 sequences and 2 Wuhan reference genomes. **(B)** Timeline of cases appearing in geographical regions. **(C)** Jaccard similarity within geographical regions. Jaccard distance was calculated between all pairs of samples in the same region. The overall median is indicated as a dotted line. The geographical regions were ordered based on the date of the first case in the region.

In order to verify whether space-time clusters were transmission clusters based on SARS-CoV-2 genome sequences, we explored the genetic heterogeneity among 172 cases, within and between space-time clusters. The evolutionary relationships among SARS-CoV-2 genomes included in different spatiotemporal clusters were first examined using a phylogenetic tree (Figure 2A). Overall, most spatiotemporal clusters did not appear as a monophyletic group on the phylogenetic tree. However, most cases in cluster #7 appeared on the same branch together, as did all cases in cluster #3 and cluster #5 that appeared at the very beginning of the outbreak, seven days or more before the peak of the epidemic curve (March 18) (Figure 2B). Similarly, the sub-clusters within the Lausanne region did not show any clear clustering on the phylogenetic tree, except for the last Lausanne cluster (cluster #16), which occurred after the lockdown (March 16).

We compared the genetic homogeneity among spatiotemporal clusters, where the genetic similarity between pairs of samples was quantified with the Jaccard similarity index. In general, intra-cluster genetic similarity was higher in rural regions than in urban areas (*p*-value < 22e-16, Wilcoxon). The genetic similarity was greater than the median in four clusters (clusters #3, #5, #7, #17) (Figure 2C). Cluster #3, #5 and #7 are early-appearing clusters that aggregated in the phylogenetic tree and showed the highest Jaccard genetic similarities. They were followed by cluster #17, which occurred in the second largest city of Vaud at the end of the first epidemic wave, after the lockdown (March 16). Clusters #11, #12 and #15 with the lowest genetic similarity appeared after March 11 (close to the peak; <7 days).

The Lausanne region, the largest urban area of Vaud, showed low similarity among cases compared to the median (Figure 2C). Although the genetic similarity of the nine clusters forming the Lausanne region remained relatively constant at low levels throughout the timeline, the genetic similarity varied over time, showing a similar pattern as other clusters with a decrease in similarity toward the peak of contaminations, and an increase back the lockdown (Supplementary Figure 3). Interestingly, Lausanne cluster #8 exhibited a significantly lower Jaccard similarity compared to cluster #7, located in the mountainous areas in the north-west of the state, even though they appeared on the same day (*p*-value < 22e-16, Wilcoxon) (Figure 2C).

### Comparison of spatiotemporal clusters and genomic groups

**Figure 3:**
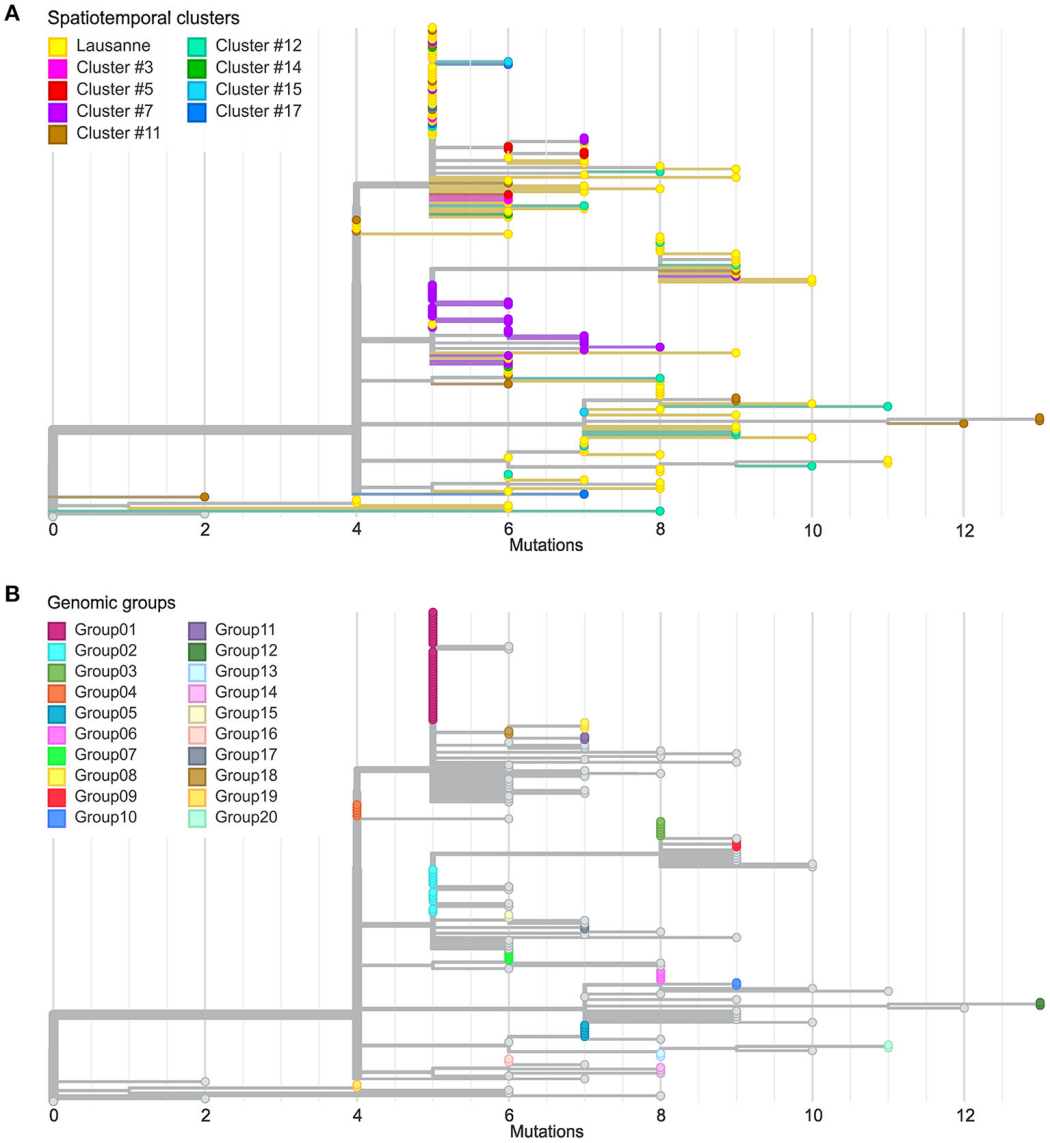
Comparison between spatiotemporal clusters and genomic groups in phylogenetic trees. **(A)** Divergence of cases in different geographical regions at SNV level. **(B)** Definition of genomic groups. Cases with identical genome sequences were assigned to a genomic group. Overall 20 genomic groups were identified with varying numbers of cases within each group. The rest of the cases have unique sequences named singletons.

**Figure 4:**
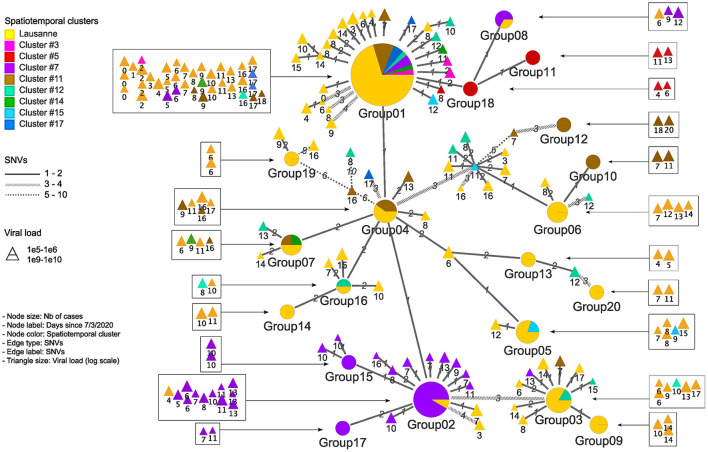
Genetic relationship among genomic groups and singletons in minimum spanning tree. Nodes and edges indicate unique sequences and SNV distance. The number of cases in genomic groups is represented by the size of the pie. Genomic groups consist of cases in different geographical regions. The triangles are single cases with their size proportional to the log value of viral load detected by qPCR. They are represented in squares according to their occurrence in time.

**Figure 5:**
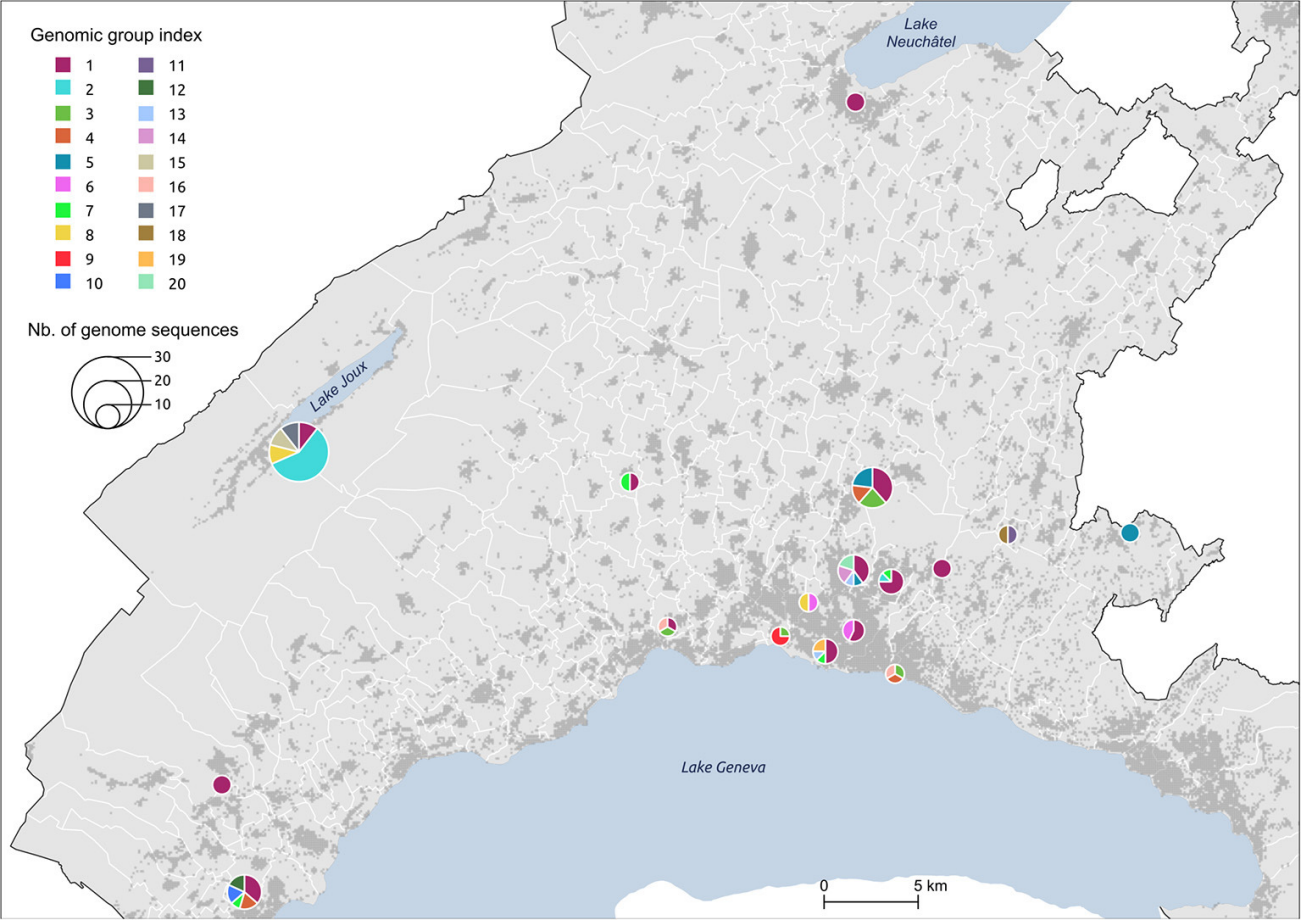
Distribution of genomic groups within clusters. The size of the circle is proportional to the number of cases.

We further investigated the genetic divergence of geographical clusters at single nucleotide variant (SNV) level (Figure 3A). The distance in SNVs compared to the Wuhan reference genome varied between 2 to 13 mutations. The first cases in the Lausanne region (in cluster #1) harbored 5 SNVs, while some later cases showed fewer mutations (2 or 4 SNVs). Among the 172 SARS-CoV-2 genomes, we identified 20 sets of cases carrying identical genomes, hereafter referred to as “genomic groups” (Figure 3B), in order to avoid confusion with geographical clusters. These 20 genomic groups include 101 of the 172 cases (group1: 37; group2: 12; group3: 6; group4 and group5: 5 each; group6 and group7: 4 each; group8 and group9: 3 each; group10-group20: 2 each). The other 71 genomes did not belong to any genomic group as they exhibited unique sequences (“singletons”). The genetic relationships among the 20 genomic groups and the 71 singletons were visualized on a minimum spanning tree network (Figure 4). This can be visualized with Figure 5, which shows the distribution of genomic groups within spatiotemporal clusters.

We identified 12 genomic groups of the 20 that were restricted to a single area (Lausanne region: 6, cluster #5: 2, cluster #7: 2, cluster #11: 2) and eight genomic groups that consisted of individuals living in two to seven different regions that always included at least one individual from the Lausanne region (Figure 4). For four spatiotemporal clusters, all cases were attributed to the same genomic group (clusters #1, #3, #15, #17) (Figure 5). We observed spatial heterogeneity within clusters, yet, unsurprisingly, cases that occurred in the same building usually shared the same genomic group (Supplementary Figure 4). The size of these multi-regional genomic groups varied between two to 37 cases. Group1 and group2 were the largest groups, with 37 and 12 cases, respectively. We investigated these groups further as there may have been superspreading events in each group. Group1 cases were split into seven geographical regions, connected to cases in the same cluster by one or two SNVs distance (Figure 4). Three of the Lausanne region cases in group1 and one case with 1 SNV distance from group1, which occurred on day 0, could represent the origin of the superspreading event that formed group1 cases. Group2 likely started with one case in the Lausanne region that was diagnosed on day 4, followed by 11 cases in spatiotemporal cluster #7 (in the mountainous north-west region). Although both group1 and group2 were identified as lineage B.1, sharing four nucleotide mutations (C241T, C3037T, C14408T, A23403G), each group was characterized by a specific mutation (Supplementary Table 2). The mutation C15324T characterized exclusively group1 and all group1-associated cases (or groups), except for group4. Likewise, the mutation A26530G featured only group2 and its neighbors (Figure 4).

## Discussion

### Congruence between two approaches in different contexts

Although the distinct use of spatiotemporal clustering and genomic-based approaches for COVID-19 management is recognized in the literature, we did not find any study investigating how the combined use of these two methods could compensate for their respective shortcomings in a surveillance context. By investigating the extent to which spatiotemporal clusters were confirmed as transmission clusters based on SARS-CoV-2 genome sequences, our results suggest that the consistency across the two methods might vary according to geographic characteristics of the area (rural/urban) and the epidemic context.

We often found less genetic similarity within clusters in urban areas compared to rural areas (*p*-value <2.2e-16, Wilcoxon). This could be explained by differences in social activities and population mobility. In rural areas, we expect many close contacts to occur among a few people from the same village, where a single introduction event might spread quickly with fewer opportunities to acquire new variants. As infected individuals in rural clusters were significantly older (*p*-value < 0.001, Wilcoxon), the genetic similarity within spatiotemporal clusters could also possibly be associated with restricted mobility of elderly people. In contrast, urban areas have numerous factors that could multiply the risk of simultaneous circulations of multiple variants, such as more frequent use of public transportation and larger places of gathering (39). Within spatiotemporal clusters, cases located in the same building were generally epidemiologically linked, as they often stemmed from within-household transmission events. Transmission in densely inhabited structures, such as cluster #16 that occurred in a migrant center after lockdown, resulted in significantly higher genetic similarity than other clusters in the Lausanne region (Supplementary Figure 3).

Moreover, the congruence between spatiotemporal and transmission clusters appeared to vary along the epidemic curve. The genetic similarity was typically higher during the lockdown and at the very beginning of the pandemic, where only a few cases were detected, than during the epidemic peak. As no study to our knowledge has examined the congruence of space-time scan and genetic clustering for SARS-CoV-2, it is difficult to interpret our findings in light of other publications. However, several studies have investigated similar research questions in the context of sexually-transmitted diseases. For example, authors found that space-time scan clustering was less successful than genetic clustering in identifying HIV-transmission patterns in small or urban HIV-endemic areas of Los Angeles County (8), while a study in the Netherlands observed a higher incidence of Hepatitis B associated with higher genetic clustering in rural areas (40). However, even if similar patterns were observed in our study, the marked differences in disease characteristics do not permit a direct comparison.

In both genomic group1 and group2, the first cases from the Lausanne region seemed to spread in many neighboring areas, including a geographically isolated area (cluster #7), showing the significant impact of urban areas and superspreading events. Genomic group1 and group2, assigned to B.1 lineage, were differentially characterized by the mutations C15324T and A26530G, respectively. First, the mutation C15324T was suspected to originate from Mulhouse (France) according to Stange et al. (23), where the first case with an identified source of infection was from a religious gathering in Mulhouse. This mutation was the main feature of that local cluster (“Basel-city”) in the early period of the first wave. Moreover, the mutation C15324T was found in other countries, mostly France and Luxembourg at considerable proportions (18.70% and 20.69% of population sequenced, respectively), but not in Italy (until 23rd March 2020). Second, the mutation A26530G was mentioned by Alteri et al. (41) as a key feature of the early Lombardy (Italy) cluster, with >90% of intra-patient prevalence circulating mid-February. It was assumed to be the origin of the subsequent transmission chain in the Lombardy region based on its small number of foreign sequences at the bases of the transmission chain. Thus, we hypothesize that superspreading events in genomic group1 and group2 might stem from secondary cases of Mulhouse and Milan outbreaks, respectively.

The major strength of the present study lies in the fine-scale resolution of the analysis, and the high-quality dataset used to investigate the interplay between genomic and spatiotemporal clustering approaches. At the beginning of the pandemic, the Institute of Microbiology of Lausanne University Hospital received all samples from Vaud state ensuring a comprehensive coverage of all cases in the area within the time frame studied here. This was rarely achieved in most other regions that commonly had multiple testing and sequencing centers, which makes it difficult to obtain an in-depth overview of the local epidemiology. However, the sampling of individuals could be biased due to untested individuals, likely leading to underestimates of superspreading events. Indeed, at the beginning of the pandemic, only symptomatic individuals were tested, although asymptomatic but contagious individuals could have contributed to the spread of the virus. Furthermore, only a portion (*n* = 172; 8%) of total positive cases were sequenced in the present study, which could affect the generalization of our results. In comparison, Bruningk et al. (42) sequenced 40% (*n* = 247) of the positive cases in the city of Basel, providing a much higher resolution but limited to a single town. As a tradeoff between the size of the study area and the sequencing density, our choice was partly dictated by the objective of comparing transmission within rural and urban settings, which is rarely done. In addition, the mobility restrictions (e.g., lockdown, homeworking, restaurants closure) and the limited genomic distances observed during the early pandemic could inflate the genetic similarity observed within spatiotemporal clusters. Novel analyses using data from successive waves might refine our findings.

### Combining genomic and spatiotemporal clustering approaches in infectious disease surveillance

Timing is a crucial factor in any surveillance system. Space-time scan statistics can be run automatically as soon as new data arrive and in near real-time using the SaTScan software (43) in batch mode. It constitutes, therefore, a powerful exploratory approach to detect high-incidence areas where authorities could prioritize cases for genome sequencing and contact tracing. The New York City Department of Health and Mental Hygiene already adopted this approach to prioritize interviews of patients and develop targeted actions for testing and prevention (7, 44). Our results suggest that one could restrict investigations to a smaller number of cases for clusters in rural areas or within the same building due to the high probability of epidemiological linkage, but also that during peak period, spatiotemporal clusters do not necessarily indicate transmission clusters. Because there are now multiple providers for COVID-19 testing, the space-time scan analysis should use newly reported infectious disease cases to regional authorities, a mandatory procedure in Switzerland. The input parameters should be fine-tuned following the recommendations from Greene et al. (7), for example, by considering the number of tests rather than the total population as the underlying at-risk population to consider changes in testing rates.

An optimal framework for infectious disease surveillance may also be complemented by other approaches. Wastewater monitoring can give a reasonable estimate of infection level and circulating variants taking into account asymptomatic patients (45), while epidemiological models can make projections about epidemic trajectories and healthcare capacity and estimate intervention scenarios (46). Incorporating data from mobility patterns using, for example, aggregated mobile phone data (21), could also improve the spatiotemporal analysis of COVID-19 dynamics, allowing for the detection of infections outside the residential neighborhood, such as at work or activity sites. Even though our study was limited to SARS-CoV-2, we could imagine a similar framework for the Monkeypox virus surveillance, where space-time scan statistics (47) and phylogeographic investigation (48) were already used to disentangle disease dynamics.

## Conclusion

**Figure 6:**
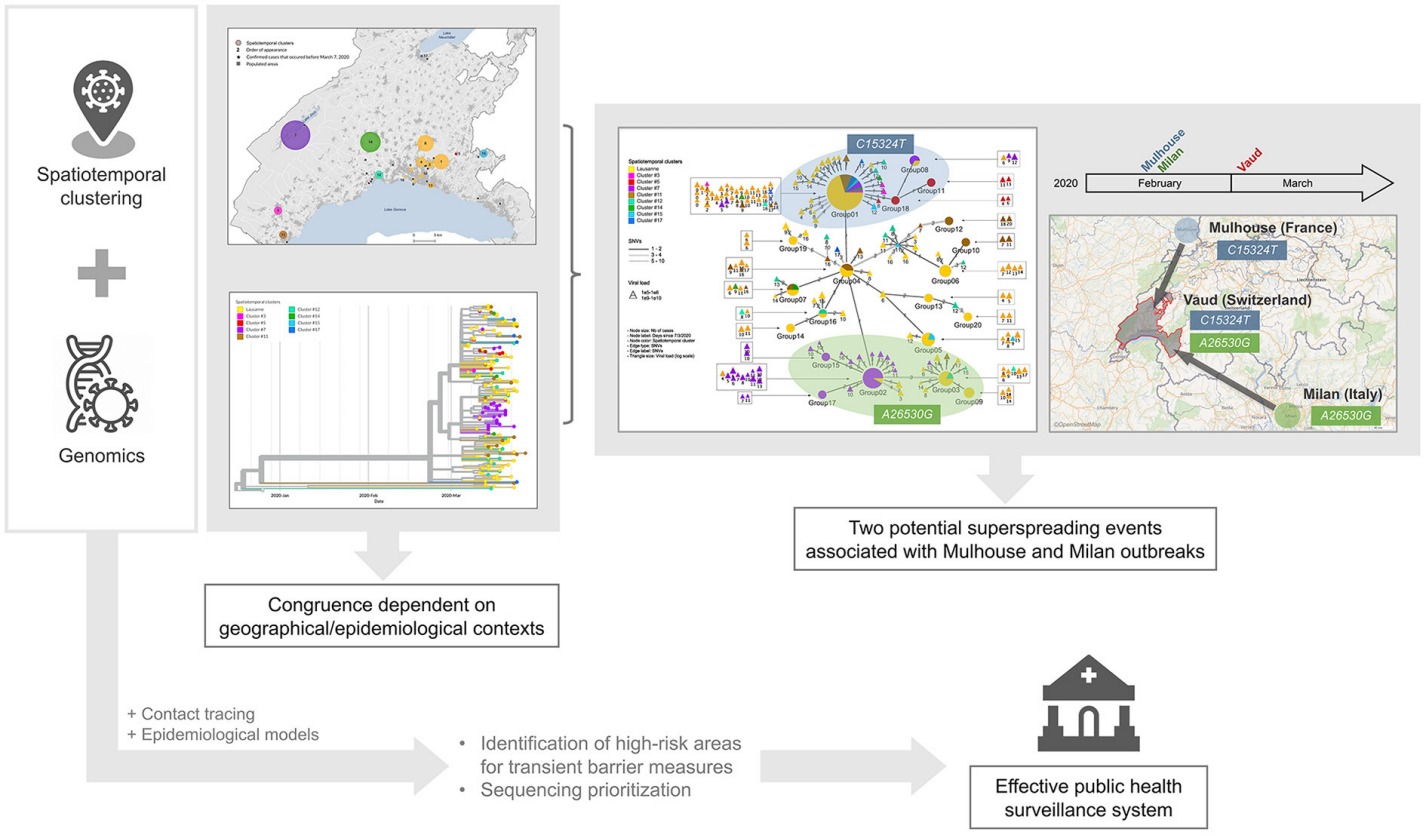
Graphical representation of findings and conclusion.

Spatiotemporal clustering and genomic approaches have been extensively used during the COVID-19 pandemic. The former approach was mainly used to identify high-incidence areas to target immediate interventions and to draw hypotheses about vulnerable populations, while the latter allowed for tracking of the origin, transmission, and evolution of the SARS-CoV-2 virus globally, and to understand host susceptibility, response, disease severity, and outcomes. In addition to the silos existing between researchers mastering each approach, spatiotemporal methods are limited by the fact that they usually consider only one source of virus transmission (i.e., the residential setting), while genomic studies require significant resources and processing time, which could delay decision-making (Supplementary Table 3). Our genomic investigation of spatiotemporal clusters showed that the clusters identified by space-time scan statistics were more likely to be epidemiologically linked in rural areas and outside the epidemic peak. In addition, we identified two potential superspreading events, characterized by specific mutations indicating their respective origins from two major outbreaks in Europe at the beginning of the pandemic. These findings suggest that we could save considerable resources and improve the efficiency of the public health surveillance system by synergizing both approaches, and prioritizing genome sequencing and contact tracing in high-incidence areas detected using spatiotemporal clustering approaches (Figure 6).

Recently, SARS-CoV-2 genomic surveillance has gradually reduced (49). Without the ability to track the virus, and while much of the world remains unvaccinated, we are unlikely to make targeted public health decisions in the face of potentially threatening new variants. We must remember the lessons from the first wave of the pandemic, when lack of data and knowledge caused societal distress, and avoid returning to such a situation by maintaining genomic-based surveillance efforts, conjointly with spatiotemporal surveillance.

## Data availability statement

The datasets presented in this study can be found in online repositories. The names of the repositories and accession numbers can be found in the article/Supplementary material.

## Ethics statement

The studies involving human participants were reviewed and approved by La Commission cantonale d'éthique de la recherche sur l'être humain (CER-VD). Written informed consent from the participants' legal guardian/next of kin was not required to participate in this study in accordance with the national legislation and the institutional requirements.

## Author contributions

SJ, SV, DJ, CB, and GG contributed to the study design. DJ and GG prepared the documentation to obtain agreement from the local ethical committee and provided the PCR data. GG obtained the funding for the study and organized with CB the SARS-CoV-2 genomes sequencing of selected samples. YC and AL analyzed all the data with the help of DD and TP. YC and AL wrote the first draft of the article. All the authors helped to improve and deepen the data analysis and all corrected the manuscript in order to obtain its final version.

## Funding

This work was supported by an unrestricted research grant in the field of diagnosis of SARS-CoV-2 infection and epidemiology of the COVID-19 pandemic from the Ferring International Center, Saint-Prex, Switzerland. Moreover, the project was partially supported by the R&D Program, Institute of Microbiology, CHUV (Center Hospitalier Universitaire Vaudois), Lausanne, Switzerland. This work was supported as a part of NCCR Microbiomes, a National Centre of Competence in Research, funded by the Swiss National Science Foundation (grant number 180575). Open access funding was provided by the University of Lausanne.

## Conflict of interest

GG has a research agreement with Becton-Dickinson on automation using the BD-Kiestra automated system as well as a research agreement with Resistell on nanotechnology to determine the antibiotic susceptibility of bacteria. In addition, Gilbert Greub is co-director of JeuPRO, a start-up distributing the card games Mykrobs and Krobs, which are two games on microbes. All these relationships with industry does not represent a direct conflict of interest on the present epidemiological work on SARS-CoV-2. The remaining authors declare that the research was conducted in the absence of any commercial or financial relationships that could be construed as a potential conflict of interest.

## Publisher's note

All claims expressed in this article are solely those of the authors and do not necessarily represent those of their affiliated organizations, or those of the publisher, the editors and the reviewers. Any product that may be evaluated in this article, or claim that may be made by its manufacturer, is not guaranteed or endorsed by the publisher.
