# Erratum: Detection of SARS-CoV-2 infection clusters: The useful combination of spatiotemporal clustering and genomic analyses

**DOI:** 10.3389/fpubh.2023.1172435

**Published:** 2023-03-03

**Authors:** Frontiers Production Office

**Affiliations:** Frontiers Media SA, Lausanne, Switzerland

**Keywords:** SARS-CoV-2, COVID-19, epidemiology, spatiotemporal cluster, genomics, public health surveillance, superspreading, genetic similarities

An omission to the funding section of the original article was made in error. The following sentence has been added: “Open access funding was provided by the University of Lausanne.”

The publisher apologizes for this mistake. The original article has been updated.

