# Supplementary material for: Detection of SARS-CoV-2 infection clusters: The useful combination of spatiotemporal clustering and genomic analyses
Source: Front Public Health. 2022 Dec 1;10:1016169. doi: 10.3389/fpubh.2022.1016169 (PMC9771593; doi:10.3389/fpubh.2022.1016169)
Supplement: Supplementary file 1 [file Data_Sheet_1.docx]

Supplementary materials

**Detection of SARS-CoV-2 infection clusters: the useful combination of spatiotemporal clustering and genomic analyses.**

Choi Yangji*, Ladoy Anaïs*, De Ridder David, Jacot Damien, Vuilleumier Séverine, Bertelli Claire, Guessous Idris, Pillonel Trestan, Joost Stéphane*, Greub Gilbert*

*contributed equally

[Figure S1: Selection of the 17 spatiotemporal clusters considered for genomic data analysis. 2](#_Toc116048210)

[Table S1: Characteristics of the individuals for whom we analyzed the SARS-CoV-2 genomic sequence (N=172). 3](#_Toc116048211)

[Figure S2: Construction of minimum spanning tree from a network of SARS-CoV-2 genomes with their pairwise SNV distance. 4](#_Toc116048212)

[Figure S3. Jaccard similarity index of 9 spatiotemporal clusters within the Lausanne region. 5](#_Toc116048213)

[Figure S4: Spatial distribution of genomic groups within spatiotemporal clusters. 6](#_Toc116048214)

[Table S2: Pangolin lineage and nucleotide sequence changes of 20 genomic groups. 9](#_Toc116048215)

[Table S3. Advantages and disadvantages of spatiotemporal clustering and genomics in surveillance. 9](#_Toc116048216)


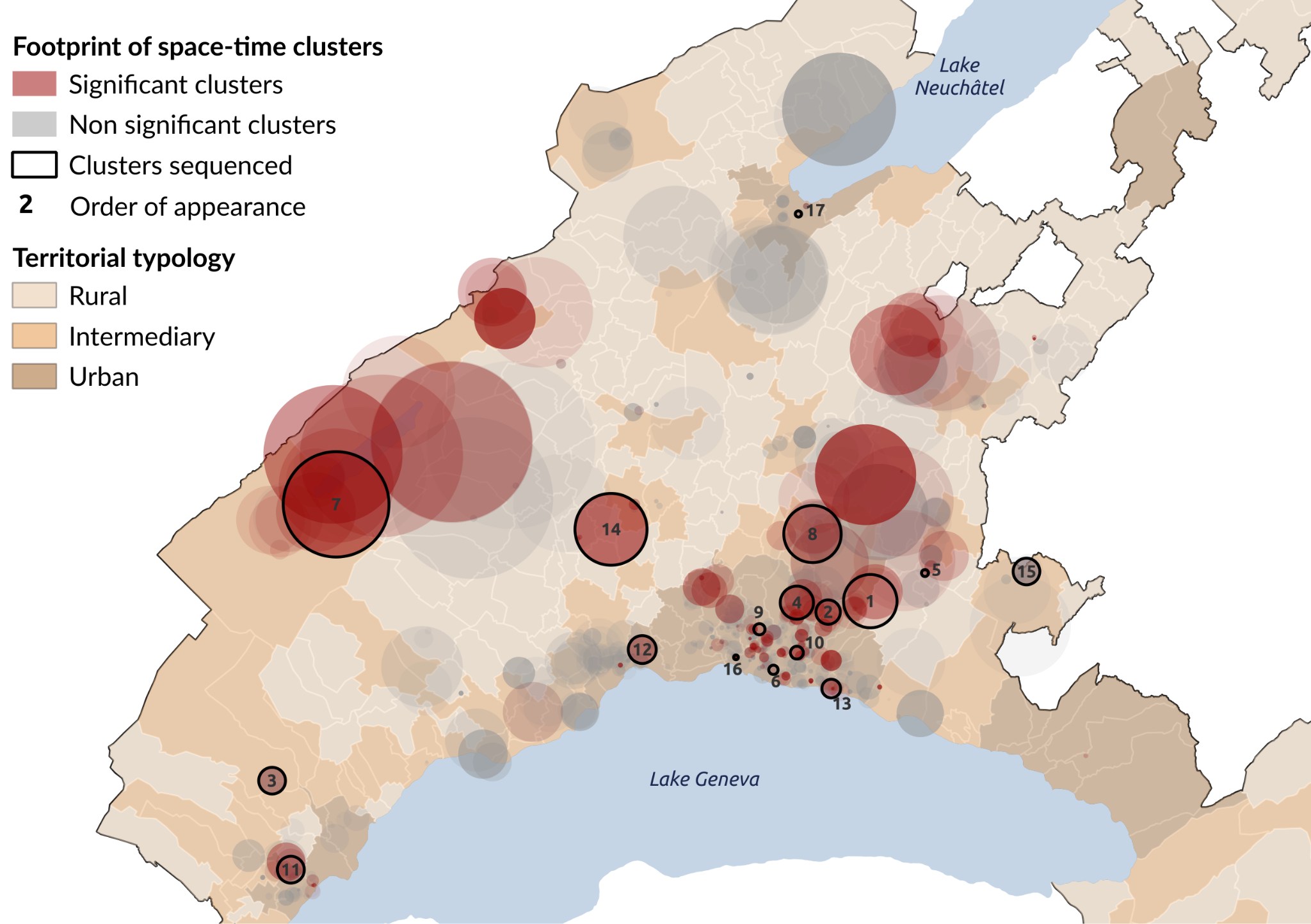


#### Figure S1: Selection of the 17 spatiotemporal clusters considered for genomic data analysis.

Overlaid on this map are all clusters of SARS-CoV-2 cases detected daily from March 2 to June 30, 2020 period using space-time scan statistics [[1]](https://paperpile.com/c/adRGem/RSbh). The 17 geographical clusters selected for genome sequencing are labeled according to their order of apparition. The clusters were selected from different urban-rural contexts that are represented here using the territorial typology developed by the Federal Statistical Office in 2012 [[2]](https://paperpile.com/c/adRGem/CTiN).

Note that in space-time scan statistics, large clusters do not necessarily correspond to a high number of cases, but rather to an unusually high relative risk of COVID-19 cases detected in this area. For a similar number of cases observed in a defined area, the relative risk is per definition lower in high density populated areas. We differentiate clusters that had a p-value < 0.05 (red) or > 0.05 (gray) based on 999 Monte-Carlo permutations.

| **Cluster** | **N** | **Age (SD)** | **Females (%)** | **Viral load (SD)** |
| --- | --- | --- | --- | --- |
| *Lausanne region* | *95* | *53.1 (18.8)* | *47 (49.5)* | *5.6e+08 (1.1e+09)* |
| #1 | 7 | 58.6 (14.8) | 2 (28.6) | 2.7e+08 (4.2e+08) |
| #2 | 10 | 47.7 (17.1) | 7 (70) | 1.2e+09 (2e+09) |
| #4 | 12 | 66.7 (17.1) | 6 (50) | 4e+08 (6.6e+08) |
| #6 | 13 | 52.9 (20.5) | 7 (53.8) | 2.5e+08 (3.8e+08) |
| #8 | 21 | 57.6 (18.3) | 13 (61.9) | 7.4e+08 (1.3e+09) |
| #9 | 3 | 55.6 (22.4) | 2 (66.7) | 1.6e+08 (1.6e+08) |
| #10 | 19 | 46.4 (16.3) | 6 (31.6) | 6.4e+08 (1.3e+09) |
| #13 | 5 | 44 (18.6) | 1 (20) | 2.7e+07 (2.4e+07) |
| #16 | 5 | 50.3 (6.83) | 3 (60) | 4.4e+08 (4.4e+08) |
| #3 | 3 | 62.1 (7.12) | 1 (33.3) | 1e+08 (1.6e+08) |
| #5 | 5 | 50.9 (26.3) | 2 (40) | 2.1e+08 (4e+08) |
| #7 | 31 | 71.6 (19.3) | 20 (64.5) | 2.7e+08 (5.9e+08) |
| #11 | 16 | 54.8 (17.3) | 8 (50) | 1.1e+09 (2.4e+09) |
| #12 | 12 | 50.7 (20.9) | 5 (41.7) | 1.1e+08 (2e+08) |
| #14 | 3 | 65.3 (9.93) | 1 (33.3) | 3.7e+08 (5.4e+08) |
| #15 | 3 | 45.8 (3.05) | 2 (66.7) | 9.7e+07 (1.2e+08) |
| #17 | 4 | 78.0 (27.1) | 4 (100) | 3.5e+07 (4.5e+07) |

#### Table S1: Characteristics of the individuals for whom we analyzed the SARS-CoV-2 genomic sequence (N=172).

Viral load is expressed as number of copies/ml.


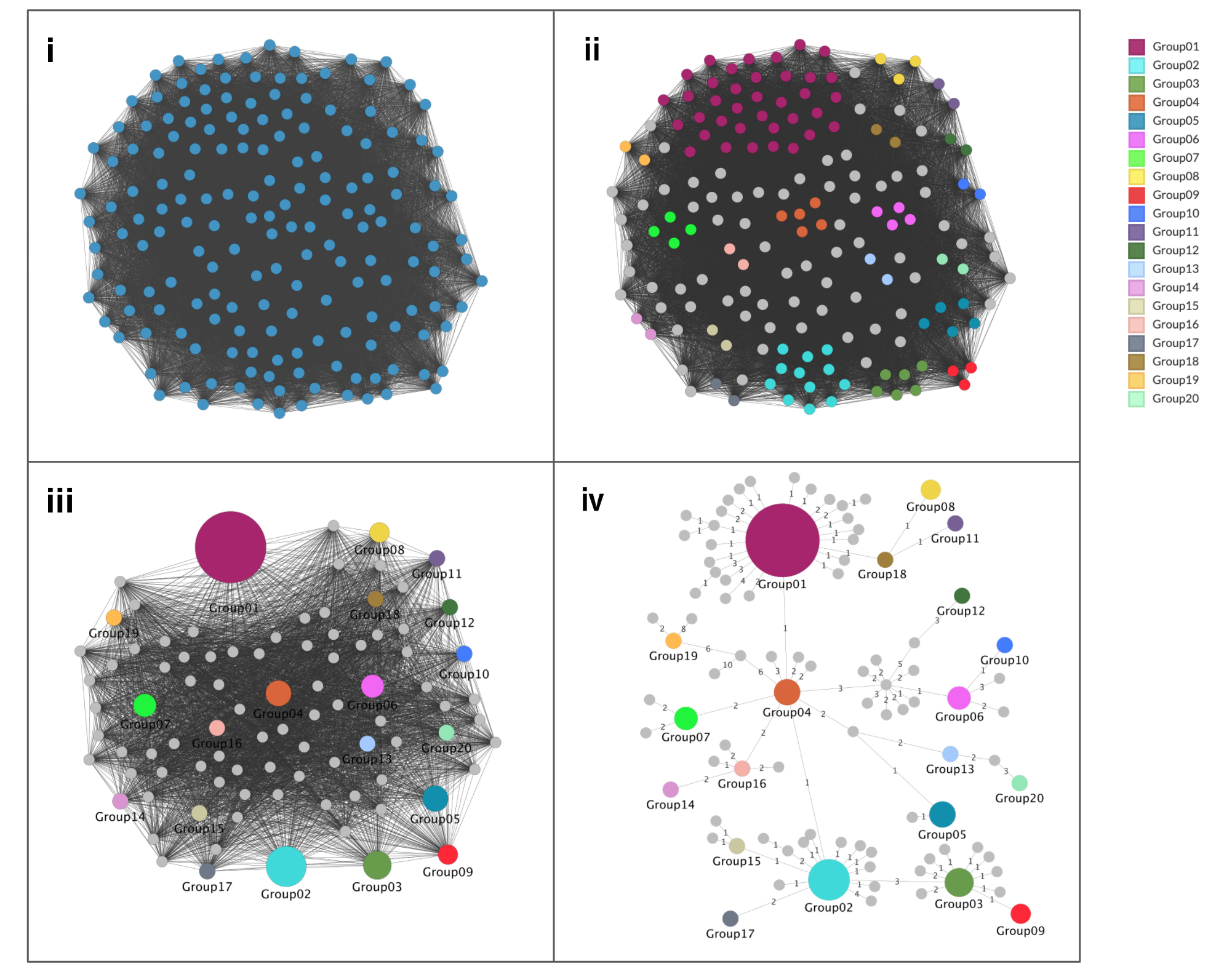


#### Figure S2: Construction of minimum spanning tree from a network of SARS-CoV-2 genomes with their pairwise SNV distance.

i. 172 sequences represented as nodes are connected with vertices indicating SNV distance. ii. 20 “genomic groups” with identical sequences are identified. iii. Sequences in the same genomic groups are merged into a single node. iv. Minimum spanning tree was built from the previous network using Prim’s algorithm, which gives the shortest path to go through all nodes.


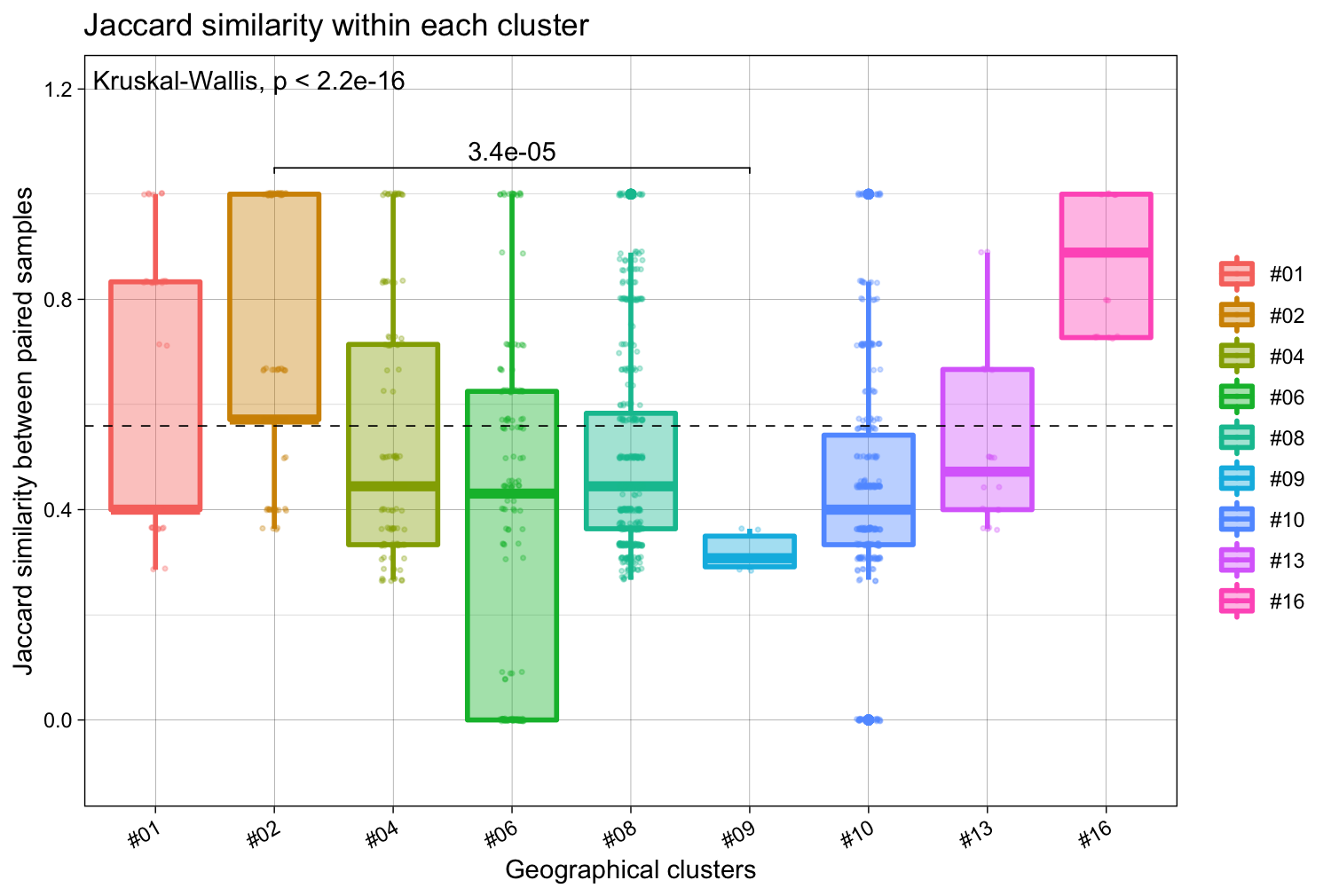


#### Figure S3. Jaccard similarity index of 9 spatiotemporal clusters within the Lausanne region.

The overall median is indicated as a dotted line. Wilcoxon test was applied for cluster #2 and #09.


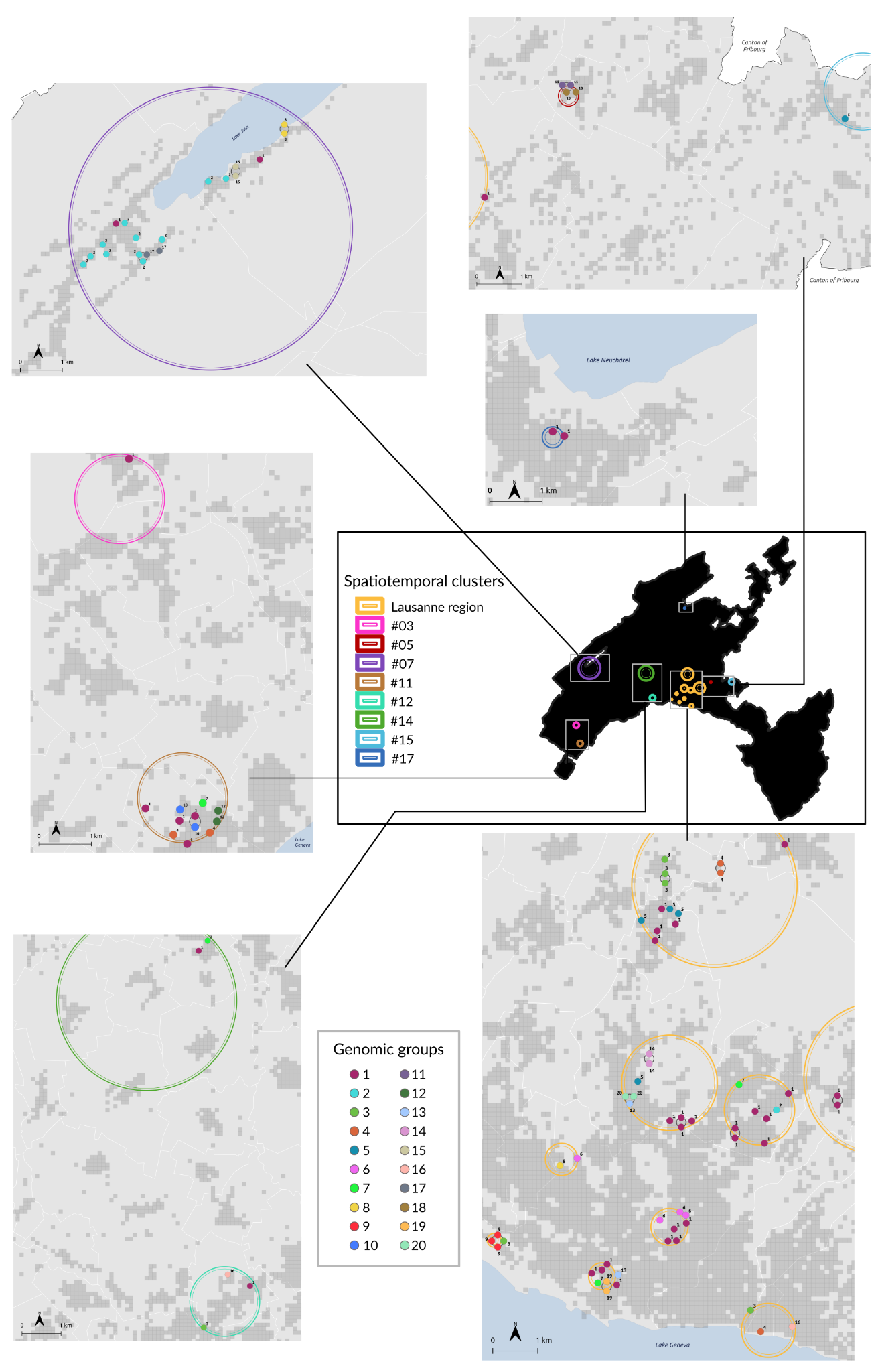


#### Figure S4: Spatial distribution of genomic groups within spatiotemporal clusters.

Each point refers to a sequenced case geocoded to the patient’s residential address, and is colored based on the genomic group to which it belongs. A small ring network represents cases that occurred in the same building. Colors of the large rings correspond to the color of the geographical clusters as summarized in the upper middle panel. Note that we randomly permuted the point’s location within clusters to maintain confidentiality.

| **Genomic groups** | **Pangolin lineage** | **Nucleotide sequence changes** |
| --- | --- | --- |
| Group01 | B.1 | C241T, C3037T, C14408T, C15324T, A23403G |
| Group02 | B.1 | C241T, C3037T, C14408T, A23403G, A26530G |
| Group03 | B.1.128 | C241T, C3037T, C8293T, C14408T, T18488C, A23403G, T24739C, A26530G |
| Group04 | B.1 | C241T, C3037T, C14408T, A23403G |
| Group05 | B.1.93 | C241T, C3037T, C13862T, C14408T, A23403G, C28045T, C28836T |
| Group06 | B.1.1 | C241T, C3037T, C14408T, T19839C, A23403G, G28881A, G28882A, G28883C |
| Group07 | B.1 | C241T, C3037T, C14408T, A20268G, A23403G, C28854T |
| Group08 | B.1 | C241T, C3037T, T14208C, C14408T, C15324T, A23403G, T24022C |
| Group09 | B.1.128 | C241T, C3037T, G7675T, C8293T, C14408T, T18488C, A23403G, T24739C, A26530G |
| Group10 | B.1.1 | C241T, C3037T, C14408T, T19839C, A23403G, G23426T, G28881A, G28882A, G28883C |
| Group11 | B.1.1 | C241T, C3037T, C4320T, T14208C, C14408T, C15324T, A23403G |
| Group12 | B.1.1.1 | C241T, C3037T, C4002T, G10097A, C13536T, C14408T, C20104T, C20946T, A23403G, C23731T, G28881A, G28882A, G28883C |
| Group13 | B.1.93 | C241T, C3037T, C13862T, C14408T, C15352T, A23403G, C28836T, G29229A |
| Group14 | B.1 | C241T, C1059T, C3037T, G11083T, C14408T, C16616T, A23403G, G25563T |
| Group15 | B.1 | C241T, C3037T, C12020T, C14408T, A23403G, A26530G |
| Group16 | B.1 | C241T, C1059T, C3037T, C14408T, A23403G, G25563T |
| Group17 | B.1 | C241T, G677A, C3037T, C11511T, C14408T, A23403G, A26530G |
| Group18 | B.1 | C241T, C3037T, T14208C, C14408T, C15324T, A23403G |
| Group19 | B | G11083T, C14805T, T17247C, G26144T |
| Group20 | B.1.93 | C241T, C3037T, T3466C, C6738T, C6781T, C13862T, C14408T, C15352T, A23403G, C28836T, G29229A |

#### Table S2: Pangolin lineage and nucleotide sequence changes of 20 genomic groups.

|  | Spatiotemporal clustering | Genomics |
| --- | --- | --- |
| Advantages | - Early detection of case clusters - Indicate where to prioritize interventions and resources allocation - Rely on already collected testing data - Low processing cost | - Identification of known and novel strains - Tracking local and intercontinental transmission - International genome sequencing databases - Availability of numerous user-friendly tools |
| Disadvantages | - Do not guarantee epidemiological linkage within clusters - Centered around the residential setting | - Considerable cost - Long turnaround time |

#### Table S3. Advantages and disadvantages of spatiotemporal clustering and genomics in surveillance.

**References**

1. [Ladoy, A., Opota, O., Carron, P.-N., Guessous, I., Vuilleumier, S., Joost, S., and Greub, G. (2021). Size and duration of COVID-19 clusters go along with a high SARS-CoV-2 viral load: A spatio-temporal investigation in Vaud state, Switzerland. Sci. Total Environ. *787*, 147483.](http://paperpile.com/b/adRGem/RSbh)

2. [Chang, S., Pierson, E., Koh, P.W., Gerardin, J., Redbird, B., Grusky, D., and Leskovec, J. (2021). Mobility network models of COVID-19 explain inequities and inform reopening. Nature *589*, 82–87.](http://paperpile.com/b/adRGem/CTiN)
